# Decreased Expression of GPER1 Gene and Protein in Goiter

**DOI:** 10.1155/2015/869431

**Published:** 2015-03-12

**Authors:** Raquel Weber, Ana Paula Santin Bertoni, Laura Walter Bessestil, Ilma Simoni Brum, Tania Weber Furlanetto

**Affiliations:** ^1^Programa de Pós Graduação em Medicina: Ciências Médicas, Universidade Federal do Rio Grande do Sul, Rua Ramiro Barcelos 2400, 90035-903 Porto Alegre, RS, Brazil; ^2^Programa de Pós Graduação em Ciências da Saúde, Universidade Federal de Ciências da Saúde de Porto Alegre, Rua Sarmento Leite 245, 90050-170 Porto Alegre, RS, Brazil; ^3^Departamento de Fisiologia, Instituto de Ciências Básicas da Saúde, Universidade Federal do Rio Grande do Sul, Rua Sarmento Leite 500, 90050-170 Porto Alegre, RS, Brazil

## Abstract

Goiter is more common in women, suggesting that estrogen could be involved in its physiopathology. The presence of classical estrogen receptors (ER*α* and ER*β*) has been described in thyroid tissue, suggesting a direct effect of estrogen on the gland. A nonclassic estrogen receptor, the G-protein-coupled estrogen receptor (GPER1), has been described recently in several tissues. However, in goiter, the presence of this receptor has not been studied yet. We investigated GPER1 gene and protein expressions in normal thyroid and goiter using reverse transcription quantitative polymerase chain reaction (RT-qPCR) and Western blot, respectively. In normal thyroid (*n* = 16) and goiter (*n* = 19), GPER1 gene was expressed in all samples, while GPER1 protein was expressed in all samples of normal thyroid (*n* = 15) but in only 72% of goiter samples (*n* = 13). When comparing GPER1 gene and protein levels in both conditions, gene expression and protein levels were higher in normal thyroid than in goiter, suggesting a role of this receptor in this condition. Further studies are needed to elucidate the role of GPER1 in normal thyroid and goiter.

## 1. Introduction

17*β*-Estradiol (E2) is a member of the family of steroid hormones which controls many aspects of mammalian physiology [1]. Although its ability to stimulate breast cell proliferation is one of E2 normal roles, it increases the risk of breast cancer [1, 2].

Classically, E2 actions are mediated upon binding on two intracellular nuclear receptors, estrogen receptor (ER) alfa and ER beta, which bind to DNA and control gene expression [3]. Like other steroid hormones, it enters passively into cells where it binds to and activates ERs. It is well known that E2 has direct and indirect effects on the thyroid, and this subject has been recently reviewed [4]. These effects could explain the larger prevalence of goiter and thyroid cancer in women [5]. Although many studies have shown the expression of ER in thyroid cancer [4], there is no study associating aggressiveness of thyroid cancer and absence of ER, as it is seen in breast cancer [6].

A novel 7-transmembrane G-protein-coupled receptor, which responds to E2 stimulation with rapid cellular signaling, including ERK activation, PI3K activation, calcium mobilization, and cAMP production, has been extensively studied [7]. This receptor was named G-protein estradiol receptor 30 (GPR30), G-protein estradiol receptor (GPER), and, more recently, G-protein estradiol receptor 1 (GPER1). It has been shown to be expressed in many estradiol-responsive tissues, and some studies have proposed a potential use of selective antagonists of GPER1 as a new targeted therapy for cancer [8, 9].

GPER1 expression was described in thyroid carcinoma cell lines [10, 11] but has not been studied yet in normal or benign thyroid tissues or cells. The knowledge of the expression of this receptor in normal and nonmalignant thyroid could be a potential tool to better understand the nongenomic effects of E2, contributing to developing targets to treatment of diseases. So, the aim of this study was to evaluate the expression of GPER1 in human normal thyroid and goiter.

## 2. Materials and Methods

### 2.1. Ethics Statement

The project was submitted to, and approved by, the Research Ethics Committee of the Hospital de Clínicas de Porto Alegre (HCPA), Porto Alegre, RS, Brazil. A written informed consent from the participants was considered unnecessary by the institutional review board of the HCPA. In accordance with the Resolution HCPA 02/97, based on the Resolution CNS 196/96 of the National Health Council, Brazil, and Guideline 9 of the International Ethical Guidelines for Biomedical Research Involving Human Subjects (CIOMS, WHO, Geneva, 1993), there was no need to obtain informed consent of the patients, because only after the surgical procedure the researchers would know if a tissue sample would be available, they would not know the identity nor have access to the files of the patients, and the tissue samples would be discharged by the pathologists, so there was no interference with these exams. All tissue samples were considered medical waste and all data was anonymized (Permit number: 12-0272).

### 2.2. Human Thyroid Tissues

Thyroid samples were obtained from 16 normal thyroid and 19 goiter patients who underwent total thyroidectomy as part of treatment for differentiated thyroid cancer in HCPA. After macroscopic and frozen sections evaluation of surgical specimens by two pathologists, normal thyroid or goiter tissue, which would be discharged, was frozen in liquid nitrogen and stored at −80°C until further processing. The pattern used to confirm the presence of goiter was a well-defined fibrous capsule with a mixture of macrofollicles and microfollicles and, in some cases, some degenerative changes such as fibrosis and hemorrhage [12].

Histological criteria used to define thyroid normal tissue and goiter were described previously [13].

### 2.3. RNA Extraction

Total RNA was isolated from thyroid tissues using Trizol Reagent (Sigma-Aldrich, St. Louis, MO, USA) and quantified using a NanoDrop ND-1000 (Thermo Fisher Scientific, Wilmington, DE) via absorbance measurements at 260 and 280 nm. Only samples that presented A260/A280 ratios between 1.80 and 2.04 were used in this study. RNA was then stored at −80°C until reverse transcription (RT) into cDNA.

### 2.4. cDNA Synthesis

cDNA was synthesized from 2 *μ*g of total RNA in a 20 *μ*L reaction using oligo-dT primers and the SuperScript III Reverse Transcriptase (Invitrogen) according to the manufacturer's guidelines. cDNA samples were stored at −20°C.

### 2.5. RT-qPCR

GPER1 and *β*-actin genes were amplified in parallel using Applied Biosystems StepOne Real-Time PCR System (Invitrogen Life Technologies) and Kit Platinum SYBR Green qPCR SuperMix-UDG (Invitrogen Life Technologies). The reaction for both genes was initiated by preheating at 95°C for 10 s, followed by 40 cycles of denaturation for 15 s at 95°C, annealing for 30 s at 58°C min, and extension for 30 s at 72°C. Primer pairs used were described previously by Maggiolini et al. for GPER1 [14] and by Souza et al. for *β*-actin [15]. The specificity of the products was verified by melting curve analysis and 2.0% agarose gel electrophoresis containing 0.025 *μ*g/mL ethidium bromide.

The GPER1 and *β*-actin quantification in samples was performed based on amplification of a standard curve with five successive tenfold dilution points of a pool of cDNA samples.

### 2.6. Western Blot Analysis

Thyroid tissues were homogenized in RIPA buffer containing 20 mM (pH 8.0) Tris-HCl, 150 mM NaCl, 5 mM EDTA, 10% glycerol, plus 1 ug/mL aprotinin, 1 ug/mL leupeptin, and 0.1 mM phenylmethylsulfonyl fluoride (PMSF). Lysates were centrifuged at 12.000 ×g for 30 min at 4°C, and supernatants were used for the assay. Protein content was measured by the Bradford assay [16], and 100 micrograms of protein was separated in a 12% SDS-polyacrylamide gel with a standard molecular weight marker (Spectra Multicolor Broad, Thermo Fisher Scientific Inc, Rockford, IL, USA). Proteins were transferred to Immobilon-P polyvinylidene difluoride (0.45 *μ*m, PVDF) blotting membrane with a semidry transfer cell (Bio-Rad Trans-Blot SD, Hercules, CA, USA). Afterwards, membranes were blocked by incubation with Tris-buffered saline containing 0.1% Tween 20 and 3% nonfat dry milk for 2 h at room temperature. Thereafter, membranes were incubated with rabbit anti-GPR30 antibodies (1 : 500, Santa Cruz Biotechnology), or with mouse anti-*α*-tubulin antibody (1 : 1.000, Invitrogen) overnight at 4°C. Membranes were then incubated in horseradish peroxidase-conjugated anti-rabbit or anti-mouse immunoglobulin (1 : 7.500, Santa Cruz Biotechnology). Antigen-antibody complexes were detected by chemiluminescence and exposed to Kodak medical X-ray processor 102 (Eastman Kodak, Rochester, NY, USA). Films were scanned and the optical density of each specific band was analyzed using the Image Station 4000MM PRO (Rochester, NY, USA).

### 2.7. Statistical Analysis

Comparisons between the two groups were performed by the Mann-Whitney test; using SPSS statistical significance was established at *P* values of <0.05.

## 3. Results

**Figure 1:**
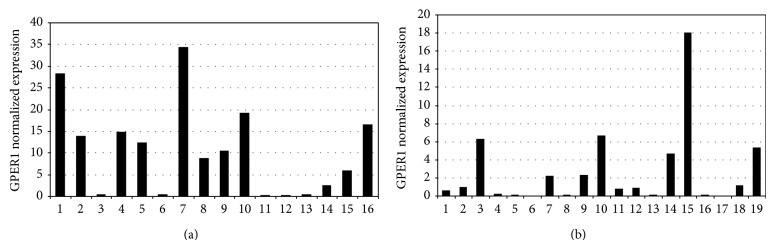
GPER1 gene expression by RT-qPCR in human thyroid. (a) Normal thyroid samples. (b) Goiter samples. *β*-actin was the reference gene. Each sample was obtained from one patient.

GPER1 mRNA was present in all samples studied (Figure 1); normal and goiter samples were obtained from the same patient in 9 cases. Results from RT-qPCR are shown as the ratio of GPER1 expression versus *β*-actin expression in arbitrary units (AU), and data are shown as median [percentile 25/percentile 75 (P25/P75)]. GPER1 mRNA was less expressed in goiter than in normal thyroid with median (P25/P75), respectively, 0.8620 (0.09/4.68) and 9.6085 (0.44/16.12).

**Figure 2:**
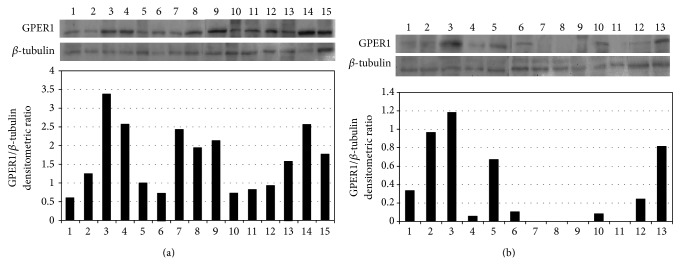
GPER1 protein expression by Western blot in human thyroid. (a) Normal samples. (b) Goiter samples. *β*-tubulin was used as loading control. Each sample was obtained from one patient.

GPER1 protein levels were evaluated in 15 samples of normal thyroid and 13 samples of goiter; normal and goiter samples were obtained from the same patient in 7 cases. The presence of a band with a molecular weight of ~38 KDa (Figures 2(a) and 2(b), upper panel) indicated the presence of GPER1. All normal thyroid samples expressed GPER1 protein, while it was not expressed in four of the goiter samples (28%). Control with *β*-tubulin in the same blot demonstrated mRNA viability in all samples. These data are shown in Figures 2(a) and 2(b).

Densitometric scanning of the 38 kDa band showed lower expression of GPER1 protein levels in goiter when compared with normal thyroid (*P* = 0.002), with median (P25/P75), respectively, 0.3332 (0.09/0.88) and 1.5804 (0.81/2.43).

## 4. Discussion

As female sexual hormones could be directly involved in the pathogenesis of multinodular goiter, we studied GPER1 gene and protein expression in 35 samples of normal thyroid and goiter. GPER1 gene expression was lower in goiter when compared to normal thyroid. Likewise, GPER1 protein levels were higher in normal thyroid than goiter, but the presence of GPER1 protein was not observed in all samples of goiter.

This was the first time GPER1 gene expression was studied in goiter; nevertheless, Gombos et al. observed previously a lower expression of this gene, as measured by high-density oligonucleotide array, confirmed by RT-qPCR, in benign and malignant thyroid tumors, when compared to normal thyroid [17]. Similarly, Kumar et al., studying papillary (*n* = 2) and follicular (*n* = 1) carcinoma cell lines, identified very low or absent levels of GPER1 gene expression [11]. On the other hand, Vivacqua et al. were able to prevent estradiol-induced transduction pathways using specific inhibitors for GPER1 in follicular (*n* = 1) and anaplastic (*n* = 2) thyroid carcinoma cell lines [10].

In other tissues, GPER1 mRNA has also been evaluated. Poola et al. reported that GPER1 mRNA levels were significantly downregulated in breast cancer tissues in comparison with their matched normal tissues. Interestingly, the receptor expression levels were lower in tumor tissues from patients who had lymph node metastasis, when with tumors without this condition [18]. Equally, GPER1 gene expression was observed to be lower in infiltrating ductal carcinoma than in nontumor mammary tissues [19] and also lower in the polycystic ovary syndrome group than in normal group [20]. On the other hand, GPER1 mRNA levels were higher in malignant than benign ovarian endometriotic cysts (EAOC) and correlated with matrix metallopeptidase 9 (MMP-9) expression, suggesting that the abnormal expression of this receptor may be involved in malignant transformation, invasion, and metastasis of EAOC [21].

Although there are no studies concerning the functional activity of GPER1 neither in normal thyroid cells nor in goiter, the lower gene and protein expression in goiter suggests a role of this gene in its pathogenesis. Other studies have shown discrepancies in GPER1 protein levels in normal and abnormal tissues. Filardo et al. demonstrated by immunohistochemistry that GPER1 was positive in all samples of normal breast, while in primary breast cancer only 42% were GPER1 positive [22]. However, in endometrial carcinoma, lung tumors, epithelial ovarian cancer, and uterine leiomyomas, the expression of this protein was higher when compared with their matched normal or benign tissues [23–26].

Although the effect of E2 in activating the growth of thyroid cells has been shown to be an action directly mediated by ER*α* [27], Manole et al. described nongenomic mechanisms mediating estradiol effect in thyroid growth [28]. The pathophysiological implications of the lower GPER1 gene and protein expressions in goiter are unknown. Nevertheless our data, although preliminary, suggest that GPER1 abnormal gene and protein expressions could be involved in the pathogenesis of goiter as either a cause or a consequence of it. Further studies, including functional experiments, could be helpful to clarify these issues.
